# “Ich bin ein Reviewer” (“I am a Reviewer”)

**DOI:** 10.1523/ENEURO.0277-16.2016

**Published:** 2016-09-15

**Authors:** Christophe Bernard

Sometimes I feel like JFK, roaming a city of free (scientific) thinking surrounded by a wall of lack of recognition at best, of hostility at worst.

The advance of knowledge relies not only on what we produce as scientists, but also on the evaluation of our work by our peers.

The review process plays an essential role in making the science that we publish stronger. We are acknowledged for the work we do as scientists, but not for the work we do as reviewers – although the review process is equally as important.

At SfN, we are proud to participate in the up-coming peer-review recognition week to recognize all that reviewers contribute to the scientific community.

We want to continually show our appreciation to our reviewers beyond this week, and have added a list of the reviewers to *eNeuro* papers in 2015 to our website that will be updated yearly. Stay tuned for more ongoing, innovative ways we will acknowledge the work of our reviewers at *eNeuro*.

Because without the reviewers volunteering their valuable time to the peer-review process, we, as scientists, would not be able to make meaningful contributions to the collective, and growing body of scientific knowledge.

So, yes, I am a scientist, but I am reviewer too, and proud of it.

